# Divergence and isolation of cryptic sympatric taxa within the annual legume *Amphicarpaea bracteata*

**DOI:** 10.1002/ece3.2134

**Published:** 2016-04-12

**Authors:** Rebecca Y. Kartzinel, Daniel Spalink, Donald M. Waller, Thomas J. Givnish

**Affiliations:** ^1^ Department of BotanyUniversity of Wisconsin‐Madison430 Lincoln DriveMadisonWisconsin53706

**Keywords:** Amphicarpy, cryptic speciation, elliptic Fourier analysis, genotyping‐by‐sequencing

## Abstract

The amphicarpic annual legume *Amphicarpaea bracteata* is unusual in producing aerial and subterranean cleistogamous flowers that always self‐fertilize and, less commonly, aerial chasmogamous flowers that outcross. Although both morphologic and genetic variants are known in this highly selfing species, debate continues over whether this variation is continuous, reflecting the segregation of standing genetic variation, or discontinuous, reflecting distinct taxa that rarely intercross. We characterized SNP variation in 128 individuals in southern Wisconsin to assess within‐ and among‐population variation at 3928 SNPs. We also assessed genotype and leaf morphology in an additional 76 individuals to connect phenotypic variation with genetic variation. Genetic variation maps onto three strongly divergent and highly inbred genetic groups showing little relation to site location. Each group has a distinct phenotype, but the divergence of these groups differs from the varietal divisions previously identified based on morphological characters. Like previous authors, we argue that the taxonomy of this species should be revised. Despite extensive sympatry, estimates of among‐group migration rates are low, and hybrid individuals were at low frequency (<2%) in our dataset. Restricted gene flow likely results from high selfing rates and partial reproductive incompatibility as evidenced by the U‐shaped distribution of pairwise *F*_ST_ values reflecting “islands” of genomic divergence. These islands may be associated with hybrid incompatibility loci that arose in allopatry. The coexistence of lineages within sites may reflect density‐dependent attack by species‐specific strains of pathogenic fungi and/or root‐nodulating bacteria specializing on distinct genotypes.

## Introduction

The rapid development of accessible molecular genotyping and sequencing methods has greatly improved our ability to catalog biodiversity (Bickford et al. 2007). In particular, genetic and genomic methods have allowed researchers to identify cryptic species that would otherwise be nearly impossible to identify based on morphology (Hebert et al. 2004; Pfenninger and Schwenk 2007; Mutanen et al. 2015). Cryptic biological species can arise in response to ecological factors without observable morphological changes. Examples of ecological speciation include insect specialization to different food plants (host race formation, e.g., Hebert et al. 2004; Blair et al. 2005) and adaptation to climatic and geological changes (e.g., Liu et al. 2013). Alternatively, populations diverging via genetic drift can accumulate genetic incompatibilities and give rise to reproductively isolated cryptic species independent of natural selection (Nei and Nozawa 2011). These genetic incompatibilities result in reduced hybrid fitness through a variety of mechanisms, including Bateson–Dobzhansky–Muller incompatibilities (fixation of different alleles in different populations at multiple loci that lead to abnormalities when recombined in hybrids), and structural differences such as inversions or translocations that interfere with meiosis in hybrids. Notably, these hybrid incompatibilities are “intrinsic” in that low fitness results from genomic factors rather than maladaptation to parental habitats (Burke and Arnold 2001; Baack et al. 2015). The acquisition of these intrinsic hybrid incompatibilities proceeds with particular efficiency in small and highly inbreeding populations due to the enhanced effects of drift when effective population sizes are small (Grundt et al. 2006; Skrede et al. 2008; Gustafsson et al. 2014).

The annual, highly selfing legume *Amphicarpaea bracteata* varies substantially in leaf morphology throughout its range, and the division of this species into infraspecific groups was unclear before genetic data were available. Two varieties are described, *A. bracteata* var. *bracteata* and var. *comosa*, based largely on leaf and stem hairiness, with *comosa* being more pubescent (Gleason and Cronquist 1963). Many taxonomic treatments do not recognize these varietal designations, however, citing high phenotypic variation and coextensive ranges of the different forms (Farwell 1924; Turner and Fearing 1964; Gleason and Cronquist 1991). More recently, genetic surveys have provided evidence for three cryptic taxa within *A. bracteata*, labeled as lineages Ia, Ib, and II (Parker 1996): lineages I and II have no alleles in common at 7 of 18 electrophoretic loci, while lineages Ia and Ib differ at only a single locus. These lineages are divergent, widespread, sympatric over a wide range, and differ in vegetative characters such as leaf shape, thickness, and hairiness. Based on DNA sequences of one plastid and two nuclear genes, lineages Ia and Ib are sister to each other, and jointly sister to lineage II, with the closely related Asian species *A. edgeworthii,* sister to all three (Parker et al. 2004). However, this genetic data does not completely support the taxonomic divisions within the species. While plants matching the description of var. *comosa* (i.e., highly pubescent) fell into lineage Ia, the treatment of var. *bracteata* was less clear, as plants in both of the remaining lineages Ib and II were sparsely pubescent yet strongly differentiated in other features (Parker 1996).

Common‐garden studies provided evidence that the phenotypic differences between varieties *bracteata* and *comosa* have a genetic basis, and offered a potential resolution to the two varieties/three lineages discrepancy by further dividing var. *bracteata* into two ecotypes defined by habitat and leaf shape: a sun‐native ecotype with wide leaflets (consistent with the phenotypes of Parker's lineage Ib), and a shade‐native ecotype with narrow leaflets (consistent with Parker's lineage II) (Callahan 1997). F3 hybrids between the morphs/lineages generally have much lower fitness than intramorph crosses, although hybrid fitness is extremely variable and can surpass that of the parents (Parker 1992). Finally, each lineage displays specific susceptibilities to a host‐specific fungal pathogen and associates with distinct nitrogen‐fixing bacteria, demonstrating that lineages have been isolated enough to evolve unique associations with pathogens and mutualists (Parker 1985, 1991, 1996; Wilkinson and Parker 1996; Wilkinson et al. 1996; Parker et al. 2004). Because each lineage hosts different strains of the fungal pathogen *Synchytrium decipiens* while resisting strains found on other lineages (Parker 1988, 1991, 1996), negative density‐dependent mortality within each lineage could facilitate local coexistence.

In short, there is strong evidence that *A. bracteata* consists of multiple cryptic taxa that are widely sympatric and have become ecologically and reproductively isolated on evolutionary timescales. Here, our goals are to use high‐resolution genomic data to (1) examine differences among the three lineages at many more loci than previously studied, (2) use such data to assess the extent of gene flow and potential reproductive isolation between lineages, and (3) assess correlations between genetic and phenotypic data to clarify the nature of the variants and the extent to which morphology supports the recognition of cryptic species within the *Amphicarpaea bracteata* complex.

## Materials and Methods

### Study species

**Figure 1:**
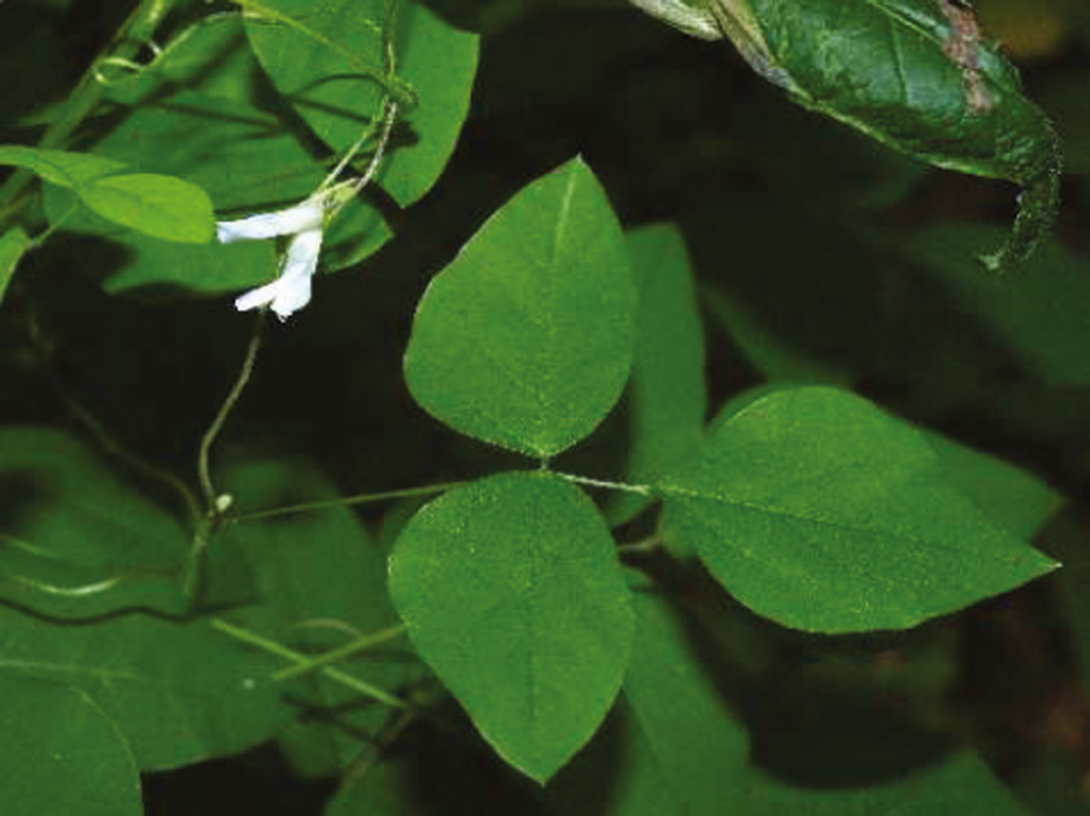
*Amphicarpaea bracteata*. Photo courtesy of Christopher Noll.

*Amphicarpaea bracteata* (Fabaceae) is an annual trailing herb, 1–5 ft long, common to moist woodland habitats of the eastern United States and southeastern Canada (Fig. 1) (Turner and Fearing 1964; Gleason and Cronquist 1991). It produces compound trifoliate leaves and, notably, three types of flowers and amphicarpic seeds that differ greatly in size (Schnee and Waller 1986). It maintains a high rate of self‐fertilization by producing two distinct types of cleistogamous flowers, both of which obligately self. Even small plants invest first in producing subterranean cleistogamous (SCL) flowers that generate large seeds (~100 mg). Larger plants then also produce aerial cleistogamous (ACL) and aerial chasmogamous (ACH) flowers, which both produce seeds of similar size (~10 mg). The large subterranean seeds produced by SCL flowers cannot disperse beyond the length of their pedicel and always germinate in the year following their production. Although the far smaller seeds produced by the aerial flowers may disperse a bit farther via myrmecochory or endozoochory, they still show restricted dispersal (Trapp 1988). They also express some dormancy, reducing germination. Because large plants only arise from large SCL seeds growing in favorable environments and because only large plants outcross via ACH flowers, lineages generally can only outcross at most every other generation.

### Sample collection

**Figure 2:**
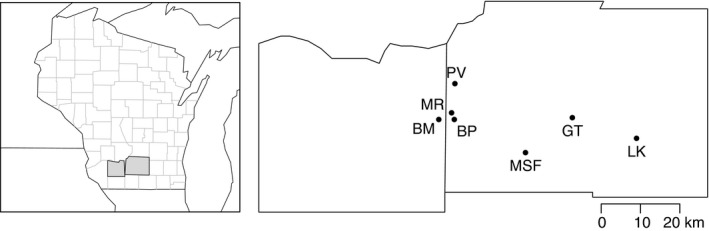
Map of study sites in southern Wisconsin.

We collected leaf tissue for DNA extraction and analysis in June–July 2013 from seven sites in southern Wisconsin (Fig. 2). Distances among sites ranged from 2 to 51 km. Leaf samples were dried on silica gel and stored at room temperature until use for genotyping‐by‐sequencing (GBS).

We revisited three sites in August 2015 to sample an additional 79 plants (BP, *n* = 26; GT, *n* = 27; and MSF, *n* = 26) for phenotyping and high resolution melt (HRM) genotyping. We chose these sites based on results from the GBS analysis, to ensure that plants from all three lineages were included in this dataset (Table S1). Upon collection, plants were placed immediately in sealed plastic bags and kept cool for transport back to the lab. We scanned each leaf using a Canon Canoscan LiDE 50 flatbed scanner and then preserved leaves in a plant press on the day they were collected. These specimens were deposited at the Wisconsin State Herbarium (WIS). We preserved additional leaf tissue from all plants on silica gel for genetic analysis.

Although our sampling was restricted to south‐central Wisconsin, it is not unlikely that we would recover the full range of genetic lineages. Parker (1996) had more extensive geographic sampling, but found that all three lineages occurred in southern Wisconsin and neighboring northeastern Illinois. Furthermore, both varieties *bracteata* and *comosa* have been reported extensively throughout the eastern United States (Turner and Fearing 1964).

### Genotyping‐by‐sequencing

Genotyping‐by‐sequencing (GBS) is in the family of reduced‐representation techniques for high‐throughput sequencing (Elshire et al. 2011; Andrews et al. 2016). Because GBS uses restriction enzymes to sample the genome, we refer to loci identified with this technique as RAD (restriction‐associated DNA) loci. We extracted DNA from the 2013 plant collections using DNeasy Plant 96 kits (Qiagen, Hilden, Germany) and then cleaned these samples with paramagnetic beads (Axyprep Mag‐PCR Clean‐up kit, Axygen, Tewksbury, MA). Libraries were prepared with ApeKI following standard GBS protocols (Elshire et al. 2011) and sequenced as 1 × 100 bp reads on the Illumina HiSeq 2500 at the University of Wisconsin‐Madison Biotechnology Center. Samples were prepared and sequenced on multiple lanes as part of 96‐plex mixed‐species libraries. To increase coverage, samples with a high proportion of missing data were resequenced in new libraries on up to four separate sequencing runs, with the reads pooled after demultiplexing. We processed raw sequences in Cutadapt (Martin 2011) to remove adapter‐contaminated reads, identified as having more than a 10‐base overlap with Illumina adapter sequences. Remaining reads were demultiplexed and filtered for quality using a sliding‐window approach (default settings) using the process_radtags program in Stacks (Catchen et al. 2013). While it is not possible to directly identify and remove PCR duplicates with this data type (Andrews et al. 2016), their effect should be reduced by Stacks algorithms that remove highly repetitive reads (‐r option in ustacks). RAD loci were built and filtered following the Stacks v1.24 de novo assembly pipeline. Default assembly parameter settings were used except that the distance between stacks (ustacks parameter *M*) was set to 5 and the distance between catalog loci (cstacks parameter *n*) was set to 3. After assembly, loci and individuals were filtered using the Stacks populations program, vcftools (Danecek et al. 2011) and custom R (R Development Core Team 2013) scripts to exclude genotypes below a log likelihood threshold of −50, individuals with >25% missing data, and loci with >10% missing data. To avoid including explicitly linked SNPs in the analysis, we only used the first SNP within each RAD locus (–write_single_snp option in the Stacks populations program).

### Population genetic analysis, hybridization, and gene flow

We used the R packages hierfstat (Goudet 2005) and pegas (Paradis 2010) to calculate basic population genetic statistics, and adegenet (Jombart 2008) to perform principal component analyses. To ensure some degree of independence, we analyzed only one SNP per RAD locus. We estimated *F*_ST_ following Weir and Cockerham (1984) in pegas.

Putative hybrids and their parental lineages were identified based on the PCA (see Results). We calculated the hybrid index (Buerkle 2005) for each putative hybrid using the R package introgress (Gompert and Buerkle 2010), along with observed heterozygosity. These statistics were compared to those from simulated F1, F2, and F3 hybrid populations, which were generated using the *hybridize* function in adegenet. One thousand F1s were generated by simulating crosses between individuals randomly sampled from each of the parental populations; 1000 F2s were then generated by selfing each F1 a single time; and 1000 F3s were generated by selfing each F2 a single time.

We used MIGRATE v.4.2 to estimate historical effective population sizes and migration rates among lineages using the coalescent (Beerli and Felsenstein 2001; Beerli 2006). For this analysis we used the full 93 bp genotypes for 905 RAD loci randomly selected from the panel used in the population genetic analysis. Putative hybrid samples were not included. The analysis was run using Bayesian inference with each lineage subsampled to *n* = 20 (with the exception of Group 1, which used the maximum *n* = 10). Twenty replicates were run with 4 chains each using a static heating scheme (temperatures of 10^6^, 3, 1.5, 1), burn‐in of 10,000 steps, and 10,000 recorded steps per chain with a sampling increment of 500. MIGRATE estimates mutation‐scaled population size, *θ *= 4N_e_ *μ*, and mutation‐scaled migration rate, *M* = m/*μ*. We then calculated the number of migrants per generation as *N*_e_ *m* = (1/4)*θ*M. The variance of these estimates depends on the number of unlinked loci (Beerli 2006). Because *A. bracteata* populations have very high selfing rates and small effective population sizes, we expect many, or even most, loci to be associated (not independent). Nevertheless, the very large number of SNP loci compensate in part for this, reducing the variance associated with these estimates to reasonably small values.

### High resolution melt genotyping

High resolution melt (HRM) genotyping uses real‐time PCR to identify variants based on small differences in the melting curves of amplicons (Liew et al. 2004). When the target region is small, a single‐nucleotide difference can sufficiently change the melting temperature to distinguish samples that are heterozygous or homozygous for alternate SNPs. HRM genotypes are identified based on melting temperature, but the inclusion of reference samples with known sequences at HRM loci allows melting temperatures to be translated to SNP genotypes.

We used HRM to screen the 2015 samples at eight RAD loci that are diagnostic for membership in one of the three genetic lineages identified in the 2013 data (see Results). The eight loci were split into two groups: four loci that separated Group 1 from Groups 2 and 3; and four that separated Groups 1 and 2 from Group 3. We selected RAD loci that were 100% diagnostic of lineages within the 2013 samples (i.e., monomorphic within groups), and additionally were appropriate for HRM primer design, with only one variable site and suitable priming sites within the 93 bp locus. We designed primers using Primer3 (Untergasser et al. 2012) (Table S3).

We used a DNeasy Plant 96 kit to extract DNA from the 79 samples obtained in 2015. We also re‐extracted 14 samples from the 2013 collection (4 each from Group 1 and Group 3, and 6 from Group 2) at the same time to use as HRM reference samples. Real‐time PCR for HRM genotyping of small amplicons was performed at the UCLA Genotyping and Sequencing Core facility using a LightCycler 480 Real Time PCR System (Roche, Branchburg, NJ) in 10 μl reaction volumes with the LightCycler 480 High Resolution Melting Master kit (Roche). We analyzed melting curves and called peaks for each sample using the LightCycler 480 software release 1.5.1.62, confirming with visual inspection.

### Leaf phenotyping and morphometric analysis

We analyzed variation in leaf morphology from the scanned images using a two‐tiered approach to determine whether we could distinguish the three genotypic lineages based only on their leaflet structure. To compare samples, we first compiled measurements from the scanned leaf images for the samples subjected to HRM genotyping using ImageJ (Schneider et al. 2012). All leaves were fully expanded and mature. Parker (1996) found that leaflet length/width ratio and leaflet hairiness are strongly associated with genotype, and we also observed variability in petiolule length among the collected samples. Therefore, we measured the following morphological traits: the length:width ratio of the terminal and right lateral leaflets (term_dim and lat_dim); the number of hairs on a 9 mm^2^ section on the abaxial surface of the terminal leaflet; and the ratio of the lengths of the terminal to the right lateral petiolule (pet_dim). Leaf hairs were counted using photographs taken with a Firefly GT200 handheld digital microscope.

Second, we conducted elliptic Fourier analysis (Zahn and Roskies 1972) on terminal and lateral leaflet shapes extracted from the scanned leaflet images using Adobe Photoshop CS3. We computed 399 harmonics for each leaflet for this analysis and conducted a PCA on the Fourier coefficients, retaining the first two axes for both the terminal and lateral leaflets. We performed these analyses using the R package Momocs (Bonhomme et al. 2014).

To compare phenotypic differences among lineage groups, we first conducted one‐way ANOVAs on leaf measurements (term_dim, lat_dim, pet_dim, and hair density) to determine which traits varied significantly among lineages. We also analyzed these variables, together with the first principal component from the elliptic Fourier analyses on the lateral leaflet shape, using linear discriminant analysis (LDA). Because the first principal component of terminal leaflet shape and term_dim were highly correlated (*r* = 0.97), we did not include terminal leaflet shape PC in this analysis. We used leave‐one‐out cross‐validation to determine the extent to which genotype can be predicted based on leaf characters.

## Results

### Genotyping‐by‐sequencing and SNP identification

In total, we obtained 313,998,762 demultiplexed reads for 141 *A. bracteata* samples for assembly in Stacks, averaging approximately 2.2M reads per individual. Stacks de novo RAD assembly with parameters *M* = 5 and *n* = 3 followed by quality filtering resulted in a final dataset of 128 individuals genotyped at 3,928 polymorphic RAD loci containing at least one biallelic SNP, with an average depth of 23 reads per locus (Table S1).

### Lineage identification

**Figure 3:**
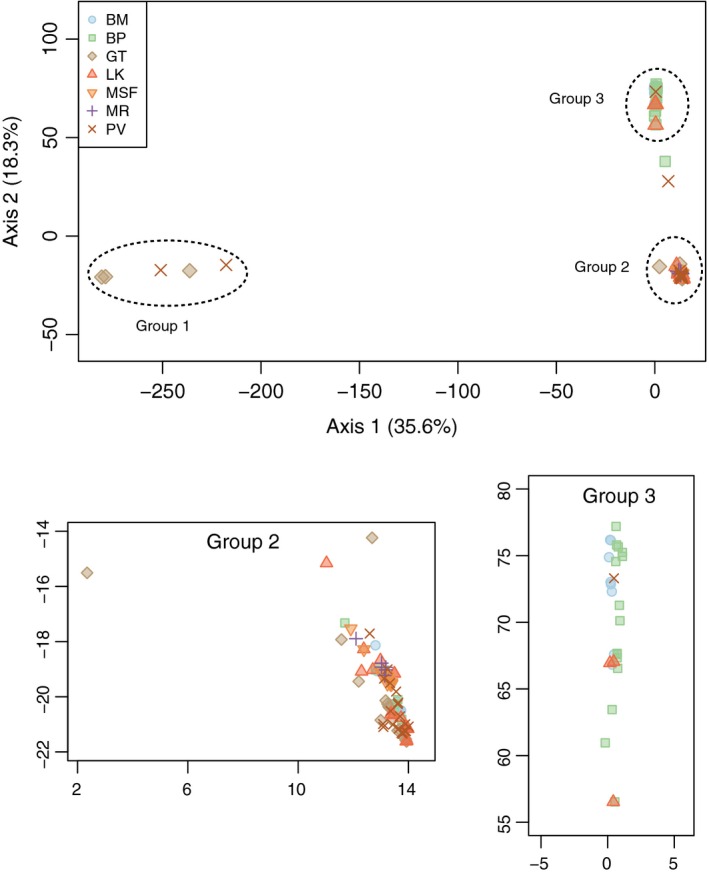
Principal components analysis based on 3928 SNPs for *Amphicarpaea bracteata* samples collected in 2013. Dashed circles denote genetic lineages/groups; panels below show detail for Groups 2 and 3.

It is clear from the PCA that site locations do not dominate the variation in the genetic data (Fig. 3). Instead, individuals separate into three distinct groups along the first two PC axes (accounting for 54% of the total variance), irrespective of sampling site. Axis 1, accounting for 35% of the variation, separates a divergent lineage (hereafter Group 1, *n* = 5) from the other individuals. Axis 2 (18.3% of the variation) divides the remaining individuals into two additional lineages (hereafter Group 2, *n* = 94; and Group 3, *n* = 29). Two individuals intermediate between Groups 2 and 3 appear to be putative hybrids. Groups were spread across multiple sites, with most sites hosting more than one group (Table S1). Individuals from putative parental lineages always occurred at sites where a hybrid was found.

Summary statistics calculated for these groups further support the contention that these represent divergent, highly homozygous lineages (Table 1a). Genic diversity is extremely low, with little allelic variation (allelic richness: 1.04–1.07 alleles/locus). Group 1 is most distinct, with a higher proportion of its alleles private to this group (35% vs. 23% for Group 2 and 17% for Group 3). This is further reflected in the genetic distances, which are high for intraspecific comparisons. Group 1 is the most divergent and equidistant from Groups 2 and 3 (Nei's genetic distance, *D*_N_ = 0.65). Group 2 and Group 3 are much more similar (*D*_N_ = 0.22).

**Table 1:** Summary statistics by (a) group, and (b) sampling site for Group 2 only. Sample sizes and number of samples per group (*n*); observed heterozygosity, *H*_*o*_; expected heterozygosity, *H*_*e*_; inbreeding coefficient, *F*_IS_; allelic richness, AR; private alleles, PA, and percent private alleles (PA/number of alleles), %PA. Hybrid individuals were not included in by‐group private allele counts

	*n*	*H*_*o*_	*H*_*e*_	*F*_IS_	AR	PA	%PA
(a) By group							
Group 1	5	0.009	0.044	–	1.04	1493	35.4
Group 2	94	0.002	0.065	–	1.07	1180	22.5
Group 3	29	0.003	0.066	–	1.06	825	17.1
(b) Group 2							
BM	11	0.002	0.045	0.961	1.04	54	1.2
BP	7	0.001	0.055	0.972	1.05	103	2.3
GT	16	0.004	0.042	0.652	1.04	168	3.8
LK	16	0.002	0.060	0.960	1.06	101	2.2
MR	6	0.001	0.040	0.971	1.04	5	0.1
MSF	18	0.001	0.006	0.886	1.01	5	0.1
PV	20	0.003	0.043	0.931	1.04	126	0.3

We additionally evaluated population‐level processes within the largest group (Group 2, with 94 samples) which was also the only group found at all seven sites (Table 1b). Heterozygosity and allelic richness are uniformly low across all sites. Rates of inbreeding are quite high, with *F*_IS_ varying from a low value of 0.65 at site GT to >0.90 at most other sites. *F*_ST_ among sites within Group 2 is 0.10.

### Hybrid analysis

The two hybrid individuals have far higher heterozygosity than their putative parental lineages, with *H*_*o*_ values at least ten times higher than the averages observed within Groups 2 and 3 (Table 2). Hybrid indices range from 0.50 (50% alleles from each group) to 0.67 (more similar to Group 3, suggesting a backcross event).

**Table 2:** Sampling site, observed heterozygosity, and hybrid index for two hybrid *Amphicarpaea bracteata* individuals. The hybrid index ranges from 0 to 1 where zero represents 100% Group 2 alleles and one represents 100% Group 3 alleles

	Site	*H*_*o*_	Hybrid index
HYB1	BP	0.070	0.67 (0.64–0.70)
HYB2	PV	0.254	0.50 (0.46–0.54)

**Figure 4:**
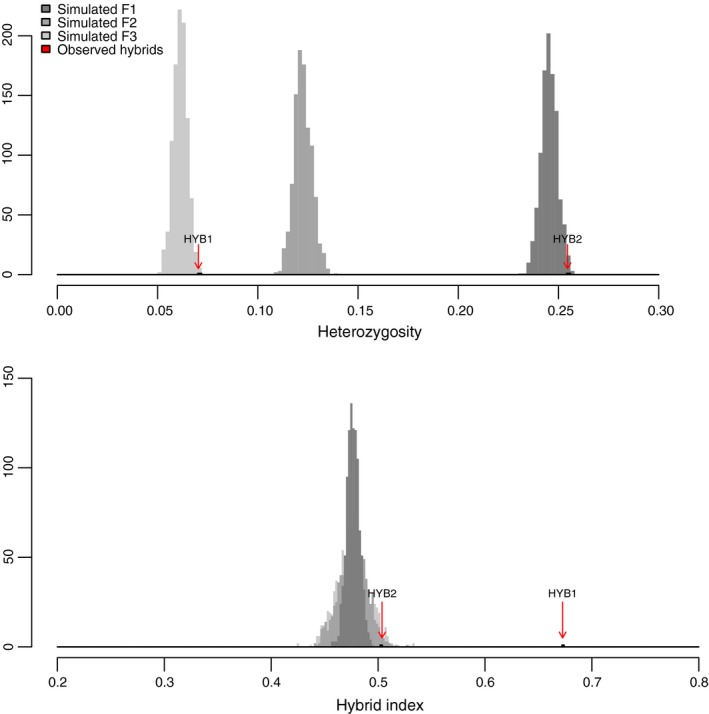
Distributions of individual observed heterozygosity (top panel) and hybrid index (bottom panel) for a simulated cross between Group 2 and Group 3. F1 (dark gray) are simulated first‐generation hybrids, and F2 (medium gray) and F3 (light gray) are after one and two generations of selfing, respectively. Values for observed hybrids found in the 2013 dataset (HYB1 and HYB2) are marked with arrows.

The simulated hybrid datasets (Fig. 4) show the heterozygosity and hybrid index expected for Group 2 x Group 3 hybrids after zero, one, and two generations of selfing (simulated F1, F2, and F3 datasets, respectively). Each generation of selfing reduces heterozygosity by 50% on average, and increases the variance of the hybrid index. The mean hybrid index is 0.48 for all simulated datasets (95% CI: 0.45–0.50), with standard deviations of 0.006 for F1, 0.013 for F2, and 0.015 for F3. Note that the simulated datasets used lineage‐level allele frequencies for Groups 2 and 3 and do not account for genetic structure among sites within lineages. As a result, the parental populations in the simulation are more diverse than in nature, and the simulated heterozygosity and hybrid index estimates may be slightly different than the values expected for natural hybrids.

Putative hybrid HYB2 closely matches the simulated F1 data in both heterozygosity and hybrid index. HYB1, however, has a higher hybrid index suggesting a backcross to Group 3, and its relatively low heterozygosity suggests that the backcross event was followed by one or more generations of selfing (arrows in Fig. 4). Alternatively, epistatic selection (Hartl and Clark 2007) could cause late‐generation selfed hybrids such as HYB1 to deviate from equal representation of parental genomes without backcrossing.

### Divergence and gene flow

Analysis with MIGRATE reveal low effective population sizes (mutation‐scaled population size, *θ*) ranging from 0.00183 to 0.00317. These values are similar to those estimated for the closely related cleistogamous soybean *Glycine max* (0.00231 and 0.00169 for wild and cultivated soybean, respectively; Lam et al. 2010). Estimated rates of historical unidirectional migration (*N*_e_ *m*) are low and asymmetric for Group 1, with immigration from Groups 2 and 3 only half the values of emigration (0.2 vs. 0.4). Groups 2 and 3 have symmetrical migration rates of approximately 0.4 (Table 3). Mutation‐scaled migration rates, *M*, with 95% confidence intervals are given in Table S2.

**Table 3:** Mutation‐scaled effective population size, *θ* (with 95% confidence intervals), and number of migrants per generation, *N*_e_ *m*, for each *Amphicarpaea bracteata* genetic group. Migration rates are unidirectional and represent movement from groups in rows to groups in columns

	*θ*	*N*_e_ *m*		
		→ Group 1	→ Group 2	→ Group 3
Group 1	0.00183 (0.00013–0.00347)	–	0.407	0.438
Group 2	0.00297 (0.00127–0.00460)	0.210	–	0.431
Group 3	0.00317 (0.00147–0.00480)	0.221	0.423	–

**Figure 5:**
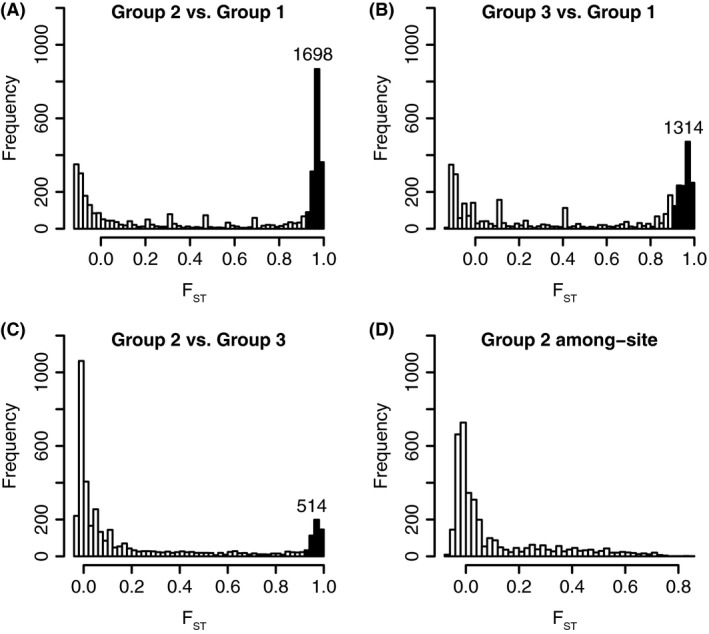
Distributions of per‐locus *F*_ST_ in all pairwise group comparisons (panels A–C) and among sites for Group 2 individuals only (panel D). Shaded bars and associated counts indicate loci with *F*_ST_ > 0.90.

The distribution of *F*_ST_ values across all loci are distinctly U‐shaped for all pairwise comparisons among lineages (Fig. 5A–C), a pattern expected in cases of divergence with gene flow. In contrast, the distribution of *F*_ST_ values calculated among sites within Group 2 shows a unimodal pattern, as expected for divergence resulting primarily from drift (Fig. 5D). Comparisons involving Group 1 (Fig. 5A, B) have a greater number of highly divergent loci (*F*_ST_ > 0.9) than the Group 2 vs. Group 3 comparison (Fig. 5C). Moreover, there is high overlap in the loci underlying the divergence of Group 2 and Group 3 from Group 1: 1066 loci have *F*_ST_ > 0.9 in both the Group 2 vs. Group 1 comparison and the Group 3 vs. Group 1 comparison. In contrast, the loci underlying the divergence of Group 1 overlap much less with those underlying the divergence of Groups 2 and 3: 254 loci with *F*_ST_ > 0.9 in both Group 2 vs. Group 1 and Group 2 vs. Group 3; 196 loci with *F*_ST_ > 0.9 in both Group 3 vs. Group 1 and Group 2 vs. Group 3.

### Lineage‐phenotype associations

We used eight diagnostic HRM loci to assign samples collected in 2015 to their genetic lineage (Table S3). Three individuals, all collected from site MSF, had HRM alleles specific to both Group 1 and Group 3 and were discarded from further analysis.

**Figure 6:**
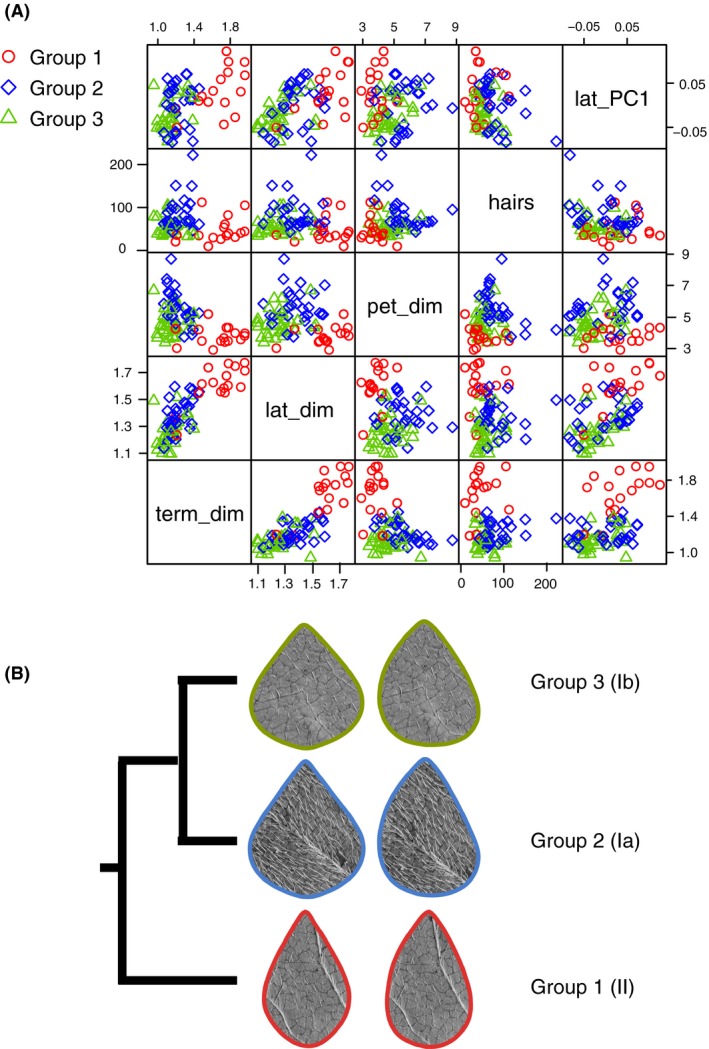
(A) Scatter plots of five morphological characters: width:length ratio for the terminal (term_dim) and lateral (lat_dim) leaflets; ratio of terminal:lateral petiolule length (pet_dim); number of hairs in a 9 mm^2^ area on the abaxial surface of the terminal leaflet (hairs); and first principal component of the elliptic Fourier analysis of the lateral leaflet (lat_PC1). (B) Phylogenetic relationships among the three groups, with an example of the typical terminal and lateral leaflet shape and hairiness. Lineage names according to Parker (1996) are given in parentheses.

Morphological analysis of the remaining 76 samples revealed extensive variation in hairiness and leaflet morphology, with a significant effect of genetic lineage (*P *<* *0.001) for all characters (Table 4, Fig. 6). Group 1, the most divergent lineage, can be separated from Groups 2 and 3 based on leaf shape, with Group 1 having narrower leaflets relative to leaflet length; and Group 2 having a significantly higher hair density than Groups 1 and 3. Cross‐validation based on the linear discriminant analysis indicates an 81.6% success rate in assigning samples to their correct lineage based on the linear model, with most misidentifications occurring in Groups 2 and 3 (Table S4).

**Table 4:** Results of ANOVA and lineage means and standard errors for four morphological traits of *Amphicarpaea bracteata*: width:length ratio for the terminal (term_dim) and right lateral (lat_dim) leaflets; ratio of terminal to right lateral petiolule length (pet_dim), and number of hairs in a 9 mm^2^ section on the abaxial surface of the terminal leaflet

	MS	*F*_2,73_	Group 1	Group 2	Group 3
term_dim	1.5194	73.36***	1.67 (0.03)^a^	1.23 (0.01)^b^	1.15 (0.01)^b^
lat_dim	0.6269	41.01***	1.61 (0.02)^a^	1.38 (0.01)^b^	1.26 (0.01)^c^
pet_dim	18.11	22.67***	3.83 (0.07)^a^	5.66 (0.13)^b^	4.66 (0.09)^c^
hairs	8776	9.879***	47.5 (3.3)^a^	84.5 (4.4)^b^	56.9 (2.3)^a^

***Signifies *P *<* *0.001. Groups with different superscript letters after mean trait values are significantly different (Tukey's HSD, *P *<* *0.05).

Our results are concordant with the phenotypes reported for the three lineages identified by Parker (1996), and our average group phenotypes also match well with described varieties and ecotypes. Group 1 matches the narrow‐leaved ecotype of *A. bracteata* var. *bracteata* (i.e., Parker's lineage II), Group 2 matches var. *comosa* (Parker's lineage Ia), and Group 3 matches the wide‐leaved ecotype of var. *bracteata* (Parker's lineage Ib).

## Discussion

*Amphicarpaea bracteata* has been described both as a single, highly variable taxon (“scarcely divisible into varieties”, Gleason and Cronquist 1991) and as a species with morphologically distinct varieties and ecotypes (Callahan 1997). Consistent with the patterns identified by Parker and others (Parker 1996; Wilkinson et al. 1996; Parker et al. 2004), we find strong evidence for three distinct but sympatric genetic lineages within *A. bracteata*. Our fine‐scale approach based on variation among 3,928 SNP markers further allowed us to clearly identify hybrid individuals and to evaluate patterns of divergence and gene flow among lineages. While individual lineages display the low diversity and inbreeding expected for a highly selfing species with limited dispersal, cross‐lineage comparisons suggest hybrid incompatibilities and divergence despite some gene flow. The phenotypic differences in leaf morphology we found among the three lineages correspond to known varieties and ecotypes (*comosa*, narrow‐leaved *bracteata* and wide‐leaved *bracteata*). We also see that taxonomic subdivisions of *A. bracteata* are not concordant with relationships among the genetic lineages.

### Three distinct taxa within *A. bracteata*

Our identification of three distinct, highly homozygous lineages within *A. bracteata* matches previous work based on 18 allozyme loci (Parker 1996) and sequence data from three nuclear and chloroplast genes (Parker et al. 2004). As with these earlier studies, we found that these lineages often co‐occur on both local and regional spatial scales: all three lineages were present within our sampling region of southern Wisconsin, and we found multiple lineages at five of our seven sampling sites. Yet despite frequent sympatry and evidence for hybridization, we find remarkably high genetic differentiation among these lineages. Leaf morphologic characters (primarily leaflet shape and hairiness) have also diverged among lineages, with Group 1 matching the narrow‐leaved ecotype of var. *bracteata*, Group 3 matching the wide‐leaved ecotype of var. *bracteata*, and Group 2 matching var. *comosa*.

Interestingly, the phenotypic differences we observe among these lineages are less clear than the genetic differences. Average phenotypes within each lineage match the taxa described within *A. bracteata*, but there is also considerable variation among individuals within lineages. Although leaf shape suffices to distinguish Group 1 from Groups 2 and 3, the measured characters fail to clearly distinguish Groups 2 and 3. Notably, Groups 2 and 3 overlap substantially in leaf hairiness, the main character used to distinguish the varieties *bracteata* and *comosa*. This overlap could reflect the presence of cryptic hybrids undetected in our HRM surveys. However, the rarity of hybrids detected using fine‐resolution GBS methods suggests instead that cryptic hybrids play only a very limited role here. This overlap in hairiness combined with similarity in leaf shape between Groups 2 and 3 will likely confound efforts to distinguish these two lineages on the basis of leaf characteristics, as seen in the rate of Group 2 – Group 3 misidentifications in our cross‐validation analysis. Thus, it is not surprising that taxonomists confronted with this variation in pubescence have hesitated to recognize the two varieties as legitimate taxa.

Our combined genetic and phenotypic data suggest that a taxonomic revision of *A. bracteata* is needed. While we did not include an outgroup in this study, a phylogeny including all three *A. bracteata* lineages, the closely related Asian species *A. edgeworthii*, and the members of the sister genus *Glycine* (*G. max* and *G. tomentella*) place the lineage corresponding to our Group 1 as basal to the other two *A. bracteata* lineages (Parker et al. 2004). Assuming we recovered and correctly identified the same *A. bracteata* lineages, we can conclude that the splitting of the species into two varieties based on pubescence is inconsistent both with the genetic data, which identifies three, not two, genetically distinct lineages; and also with the phenotypic data, which shows that the major morphological difference concordant with the deepest division among the three lineages is leaf shape (narrow vs. wide leaflets) rather than leaf pubescence.

### Isolation, divergence, and gene flow

Parker (1991, 1996) proposed several possible mechanisms that could maintain distinct lineages of *A. bracteata* in sympatry: (1) limited outcrossing due to the high selfing rate; (2) assortative mating during outcross events, perhaps due to differences in flower color among lineages; and (3) postmating barriers that reduce the fitness of hybrid individuals. The mating system of amphicarpic plants certainly limits outcrossing events, as evidenced by the high homozygosity and inbreeding coefficients documented here and previously (Parker 1988). The role of assortative mating has not yet been evaluated in this species, but our estimates of gene flow suggest that assortative mating does not strongly influence mating patterns, at least in the region studied here. Our estimates of historical migration rates among lineages are low, but this likely reflects limited opportunities for outcrossing and seed and pollen dispersal rather than assortative mating. Furthermore, the detection of hybrid individuals in our dataset, albeit at low frequency (2 in 130 samples), indicates that hybridization events occur at a non‐negligible rate.

Postmating reproductive isolation could, however, contribute to the divergence of the three lineages. The U‐shaped distribution of genome‐wide *F*_ST_ values in cross‐lineage comparisons suggest that there are some regions of the genome under negative selection in hybrids, creating “islands” of genomic divergence at loci associated with reproductive isolation with other regions more free to introgress. Support for reproductive isolation in *A. bracteata* lineages also comes from previous work finding evidence for strong outbreeding depression in crosses between different lineages (Parker 1992). Because outbreeding depression was observed in a common‐garden setting, reduced hybrid fitness must reflect intrinsic genetic incompatibilities arising in recombinant genotypes (Burke and Arnold 2001) rather than the maladaptation of intermediate hybrid phenotypes to parental habitats (Rieseberg et al. 1999; Moyle and Graham 2005). However, without data on the performance of hybrids in natural environments, we cannot rule out environmental maladaptation as a factor in hybrid fitness. Callahan (1997) has presented evidence that the robust and hairy variety *comosa* is adapted to sunny habitats whereas variety *bracteata* includes both a shade‐native ecotype with thick, narrow leaves and a sun‐native ecotype with broader leaves.

Although the observed pattern of genome‐wide divergence is consistent with expectations for sympatric speciation (Savolainen et al. 2006; Baack et al. 2015), this is not likely the case for *A. bracteata*. Regardless of the nature of the underlying genetic incompatibilities (e.g., Bateson–Dobzhansky–Muller incompatibilities or chromosomal rearrangements; Rieseberg and Willis 2007), it is difficult to imagine a scenario where they would have arisen within the current sympatric, widespread distribution of all three lineages. We propose instead that the lineages may have initially diverged and acquired sterility barriers in allopatry, followed by a period of range expansion causing some hybridization and gene flow in sympatry. A more extensive survey might succeed in reconstructing the phylogeographic history of these lineages to test this hypothesis. The more pubescent var. *comosa* is reportedly more common in western parts of its range (Gleason and Cronquist 1963; Turner and Fearing 1964), suggesting that the different lineages may have expanded out of different areas of eastern North America. The high genetic similarity of individuals within a lineage across large distances (~50 km in this study but >1000 km in others; Parker 1996) despite highly restricted dispersal is consistent with range expansion after a genetic bottleneck that eliminated most within‐lineage variation. The evolution of genetic incompatibilities in allopatry may have been facilitated by *A. bracteata*'s colonizing life history and inbred mating system promoting bottlenecks and consequent small population sizes and drift (Skrede et al. 2008; Foxe et al. 2009; Wright et al. 2013; Gustafsson et al. 2014).

### Lineage coexistence

Because populations often include multiple lineages, we face the interesting ecological question of how they coexist. The leaf differences suggest possible local adaptation to different microhabitats. Variety *comosa* is putatively sun‐adapted, while narrow‐ and wide‐leaved ecotypes of var. *bracteata* are found in shadier and sunnier micro‐habitats, respectively (Callahan 1997). However, this earlier study identified the varieties and ecotypes based on leaf morphology. Given the overlap in phenotype among genetic lineages, further work is needed to determine whether local adaptation is occurring at the lineage level. Restricted seed dispersal may also reduce the extent to which different lineages overlap, even at local scales, as median dispersal distances are estimated at only 1.5 m for aerial seeds and <1 m for subterranean seeds (Trapp 1988). However, local geographic differentiation seems unlikely to account for lineage differences given that they often co‐occur.

Differential interactions with mutualists and pathogens should also be investigated for their possible role in facilitating local coexistence. Each lineage hosts specific strains of the fungal pathogen *Synchytrium decipiens*, while showing resistance to strains found on other lineages even within the same population (Parker 1988, 1991, 1996). This could facilitate coexistence by favoring the minority taxon within any given site via negative frequency dependence, preventing any one lineage from dominating the site. Lineages also vary in their compatibility with different specific genotypes of root nodulating bacteria in the genus *Bradyrhizobium*. Two lineages (Parker's Ib and II, corresponding to our Groups 3 and 1) are specialists that only associate with distinct *Bradyrhizobium* genotypes. The other lineage (Ia, Group 2) is a symbiont generalist, an apparently derived character that could increase plant fitness and promote competitive exclusion in some situations (Wilkinson and Parker 1996; Wilkinson et al. 1996; Parker et al. 2004).

In highly selfing species such as *A. bracteata*, however, single traits like selection of symbiotic associates or disease resistance may not accurately predict the relative fitness of different genotypes in natural populations. As lineages become more inbred and homozygous, associations tend to build up even among unlinked loci as linkage disequilibrium rises (Brown and Feldman 1981; Foltz et al. 1982). This could allow associations among correlated suites of ecologically relevant traits to increase. However, forces of drift and fixation also increase in small populations (Whitlock and Barton 1997), increasing the number of associations between quantitative and nonadaptive or deleterious loci as well (Paland and Schmid 2003). Thus, even morphological characters that strongly influence fitness could evolve nonadaptively in response to selection on associated loci (e.g., Parker 1991; Porcher et al. 2006).

Multiple lines of evidence lead us to conclude that *Amphicarpaea bracteata*, long identified as a highly polymorphic species, is actually composed of three widespread, cryptic taxa. These taxa remain distinct despite their frequent co‐occurrence at local scales and documented instances of hybridization. This leads us to conclude that selection, plus possible limits on reproductive compatibility, is acting to maintain these three lineages. Given these genetic and morphological differences, we second Parker's (1996) call for a taxonomic revision of infraspecific divisions within the species. The overlap in phenotypes despite strong genetic divergence, however, will complicate efforts to positively identify lineages in the field. Further work is thus needed to identify other possible lineage‐diagnostic traits and judge whether the lineages are distinct enough to warrant species‐level designations.

## Conflict of Interest

None declared.

## Data Accessibility

- GBS data are available at the NCBI Sequence Read Archive, accession no. SRP072229.
- HRM genotypes and leaf images are deposited at Dryad doi:10.5061/dryad.12g7j.
- Phenotyped specimens are deposited at the Wisconsin State Herbarium (WIS).

## Supporting information


**Table S1.** Sample sizes for each site. Sample sizes for each lineage (Group 1, Group 2, Group 3, and hybrids) within sites are given in parentheses.
**Table S2.** Mutation‐scaled migration rate, *M* (= m/*μ*), with 95% confidence intervals for each *Amphicarpatea bracteata* genetic group.
**Table S3.** High resolution melt (HRM) primers and alleles used to assign samples to genetic groups.
**Table S4.** Results of leave‐one‐out cross‐validation analysis predicting lineage membership.
