# Supplementary material for: Divergence and isolation of cryptic sympatric taxa within the annual legume Amphicarpaea bracteata
Source: Ecol Evol. 2016 Apr 12;6(10):3367–79. doi: 10.1002/ece3.2134 (PMC4833626; doi:10.1002/ece3.2134)
Supplement: Supplementary file 1 — Table S1. Sample sizes for each site. Sample sizes for each lineage (Group 1, Group 2, Group 3, and hybrids) within sites are given in parentheses. Table S2. Mutation‐scaled migration rate, M (= m/μ), with 95% confidence intervals for each Amphicarpatea bracteata genetic group. Table S3. High resolution melt (HRM) primers and alleles used to assign samples to genetic groups. Table S4. Results of leave‐one‐out cross‐validation analysis predicting lineage membership. [file ECE3-6-3367-s001.docx]

**Supplemental Materials**

Table S1. Sample sizes for each site. Sample sizes for each lineage (Group 1, Group 2, Group 3, and hybrids) within sites are given in parentheses.

| **Site** | **n (G1/G2/G3/HYB)** |
| --- | --- |
| BM | 19 (0/11/8/0) |
| BP | 23 (0/7/15/1) |
| GT | 19 (3/16/0/0) |
| LK | 19 (0/16/3/0) |
| MR | 6 (0/6/0/0) |
| MSF | 18 (0/18/0/0) |
| PV | 24 (2/20/1/1) |

Table S2. Mutation-scaled migration rate, M (= m/μ), with 95% confidence intervals for each *Amphicarpatea bracteata* genetic group. Migration rates are directional and represent movement from groups in rows to groups in columns.

|  | -> Group 1 | -> Group 2 | -> Group 3 |
| --- | --- | --- | --- |
| Group 1 | -- | 548.3  (524.0 - 571.3) | 553.0  (528.7 - 578.7) |
| Group 2 | 458.3  (430.0-486.7) | -- | 543.7  (507.3 - 575.3) |
| Group 3 | 483.7  (451.3 - 509.3) | 570.3  (541.3 - 600.7) | -- |

Table S3. High resolution melt (HRM) primers and alleles used to assign samples to genetic groups. Loci are diagnostic for identification of Group 1 (G1 allele) from Groups 2 and 3 (G2G3 allele), or for the identification of Groups 1 and 2 (G1G2 allele) from Group 3 (G3 allele). Expected melting temperatures for alleles are given based on homozygous reference samples.

|  |  | Expected melting temperature (°C) | | | |
| --- | --- | --- | --- | --- | --- |
| Locus | Primer sequence (5'-3') | G1 | G2G3 | G1G2 | G3 |
| CLocus_6927 | F: ATTTCCCCATGTGAGCTCGC  R: TGCATGTGGAAAATTGAAAAGCA | -- | -- | 74.58-74.64 | 73.64-73.72 |
| CLocus_12985 | F: AGCTGGAGATGGTTTGATGGA  R: TCCCTACTGAGAAGACTTGACT | -- | -- | 75.87-75.99 | 74.99-75.37 |
| CLocus_78121 | F: TGCGAGAGTTCTGTGAGAGG  R: AGCCACTTCCCCTAACAACA | -- | -- | 79.04-79.13 | 79.54-79.65 |
| CLocus_81260 | F: CAAGAAAAACCCCAGGTGCG  R: CGTTAAGGTGGTGCAGTGGA | -- | -- | 85.30-85.74 | 84.53 |
| CLocus_30542 | F: AGATGGGCTCACTTGGTTGG  R: TGGAGGAAGCATTTGACTTCTCA | 77.81-77.87 | 78.42-78.50 | -- | -- |
| CLocus_42808 | F: TGCTGTGAAGGTATCTGGAGT  R: TAGCACGCACAACAGGTTCT | 74.51-74.67 | 75.56-75.69 | -- | -- |
| CLocus_48657 | F: GCAACTCTGTGGGACTCTCC  R: TCTACGCGCCCTGATATTGC | 77.68-77.84 | 76.85-76.95 | -- | -- |
| CLocus_59650 | F: CTCCCATTAGGTGTGCCTGG  R: TTCAACAAACTGCGGGAGGA | 82.22-82.29 | 82.88-83.02 | -- | -- |

Table S4. Results of leave-one-out cross-validation analysis predicting lineage membership. Values show the number of samples assigned to each lineage based on leaf phenotype (in columns) and their *a priori* group assignment based on genotype (in rows). 82% of samples were correctly assigned to their lineages based on phenotype alone.

|  | Group 1 | Group 2 | Group 3 |
| --- | --- | --- | --- |
| Group 1 | 13 | 1 | 2 |
| Group 2 | 0 | 24 | 4 |
| Group 3 | 1 | 6 | 25 |
